# Supplementary material for: Modeling glioblastoma heterogeneity as a dynamic network of cell states
Source: Mol Syst Biol. 2021 Sep 16;17(9):e10105. doi: 10.15252/msb.202010105 (PMC8444284; doi:10.15252/msb.202010105)
Supplement: Supplementary file 6 — Source Data for Figure 5 [file MSB-17-e10105-s004.zip › Figure5A_sourcedata/GSEA_3017/nosigdb_stateF.GseaPreranked.1622795015644/RICHARDS_NOWAKOWSKI_SCIENCE_2017_MURAL_UPREG.html]

Details for gene set RICHARDS\_NOWAKOWSKI\_SCIENCE\_2017\_MURAL\_UPREG[GSEA]

|  || Dataset | state33017 |
| Phenotype | NoPhenotypeAvailable |
| Upregulated in class | na\_pos |
| GeneSet | RICHARDS\_NOWAKOWSKI\_SCIENCE\_2017\_MURAL\_UPREG |
| Enrichment Score (ES) | 0.39993748 |
| Normalized Enrichment Score (NES) | 2.8780172 |
| Nominal p-value | 0.0 |
| FDR q-value | 0.0 |
| FWER p-Value | 0.0 |
Table: GSEA Results Summary

  

Fig 1: Enrichment plot: RICHARDS\_NOWAKOWSKI\_SCIENCE\_2017\_MURAL\_UPREG      
 Profile of the Running ES Score & Positions of GeneSet Members on the Rank Ordered List

  

| PROBE | GENE SYMBOL | GENE\_TITLE | RANK IN GENE LIST | RANK METRIC SCORE | RUNNING ES | CORE ENRICHMENT || 1 | NEAT1 |  |  | 0 | 2.864 | 0.0291 | Yes |
| 2 | SPARCL1 |  |  | 6 | 1.804 | 0.0451 | Yes |
| 3 | ATP1B2 |  |  | 9 | 1.759 | 0.0620 | Yes |
| 4 | SLC1A3 |  |  | 26 | 1.058 | 0.0649 | Yes |
| 5 | CPE |  |  | 29 | 1.046 | 0.0746 | Yes |
| 6 | ITPR2 |  |  | 34 | 1.024 | 0.0830 | Yes |
| 7 | PLTP |  |  | 35 | 1.020 | 0.0934 | Yes |
| 8 | TNFRSF21 |  |  | 36 | 1.016 | 0.1037 | Yes |
| 9 | DKK3 |  |  | 37 | 0.994 | 0.1139 | Yes |
| 10 | IFITM3 |  |  | 38 | 0.987 | 0.1239 | Yes |
| 11 | F3 |  |  | 41 | 0.978 | 0.1329 | Yes |
| 12 | A2M |  |  | 42 | 0.974 | 0.1428 | Yes |
| 13 | SPARC |  |  | 52 | 0.929 | 0.1478 | Yes |
| 14 | SESN3 |  |  | 56 | 0.908 | 0.1556 | Yes |
| 15 | LPL |  |  | 59 | 0.874 | 0.1635 | Yes |
| 16 | ITGB5 |  |  | 80 | 0.803 | 0.1619 | Yes |
| 17 | PON2 |  |  | 93 | 0.764 | 0.1638 | Yes |
| 18 | IFITM2 |  |  | 103 | 0.749 | 0.1670 | Yes |
| 19 | B2M |  |  | 110 | 0.736 | 0.1716 | Yes |
| 20 | PROS1 |  |  | 123 | 0.708 | 0.1729 | Yes |
| 21 | BTG2 |  |  | 124 | 0.708 | 0.1801 | Yes |
| 22 | TSPAN7 |  |  | 148 | 0.671 | 0.1757 | Yes |
| 23 | ITPR1 |  |  | 149 | 0.667 | 0.1825 | Yes |
| 24 | KCNE4 |  |  | 150 | 0.666 | 0.1892 | Yes |
| 25 | ITM2C |  |  | 160 | 0.649 | 0.1914 | Yes |
| 26 | TOB1 |  |  | 161 | 0.646 | 0.1980 | Yes |
| 27 | IGFBP7 |  |  | 164 | 0.643 | 0.2036 | Yes |
| 28 | CDH6 |  |  | 166 | 0.635 | 0.2095 | Yes |
| 29 | MTUS1 |  |  | 178 | 0.614 | 0.2104 | Yes |
| 30 | ST3GAL5 |  |  | 180 | 0.612 | 0.2162 | Yes |
| 31 | ITM2B |  |  | 181 | 0.610 | 0.2224 | Yes |
| 32 | NINJ1 |  |  | 182 | 0.610 | 0.2286 | Yes |
| 33 | PLOD2 |  |  | 183 | 0.607 | 0.2347 | Yes |
| 34 | CEBPB |  |  | 190 | 0.601 | 0.2379 | Yes |
| 35 | CD44 |  |  | 191 | 0.600 | 0.2440 | Yes |
| 36 | TIMP2 |  |  | 196 | 0.590 | 0.2481 | Yes |
| 37 | COL5A2 |  |  | 199 | 0.589 | 0.2531 | Yes |
| 38 | APOBEC3C |  |  | 224 | 0.572 | 0.2471 | Yes |
| 39 | SLC2A3 |  |  | 228 | 0.569 | 0.2515 | Yes |
| 40 | PSMB8 |  |  | 230 | 0.568 | 0.2567 | Yes |
| 41 | CEBPD |  |  | 234 | 0.566 | 0.2610 | Yes |
| 42 | CA2 |  |  | 243 | 0.558 | 0.2628 | Yes |
| 43 | ENDOD1 |  |  | 262 | 0.543 | 0.2595 | Yes |
| 44 | PKIG |  |  | 290 | 0.517 | 0.2516 | Yes |
| 45 | ASAH1 |  |  | 293 | 0.515 | 0.2558 | Yes |
| 46 | NUPR1 |  |  | 295 | 0.513 | 0.2606 | Yes |
| 47 | ARHGAP42 |  |  | 297 | 0.511 | 0.2653 | Yes |
| 48 | ATP6V0E1 |  |  | 303 | 0.510 | 0.2680 | Yes |
| 49 | TRIM5 |  |  | 305 | 0.508 | 0.2727 | Yes |
| 50 | GSTK1 |  |  | 312 | 0.503 | 0.2749 | Yes |
| 51 | HES1 |  |  | 320 | 0.501 | 0.2765 | Yes |
| 52 | IL6ST |  |  | 321 | 0.500 | 0.2816 | Yes |
| 53 | COBLL1 |  |  | 327 | 0.497 | 0.2842 | Yes |
| 54 | SEMA5A |  |  | 336 | 0.494 | 0.2854 | Yes |
| 55 | VAMP5 |  |  | 341 | 0.491 | 0.2884 | Yes |
| 56 | COL4A1 |  |  | 347 | 0.489 | 0.2909 | Yes |
| 57 | SPTBN1 |  |  | 349 | 0.487 | 0.2954 | Yes |
| 58 | CYSTM1 |  |  | 358 | 0.484 | 0.2964 | Yes |
| 59 | CADM4 |  |  | 368 | 0.482 | 0.2969 | Yes |
| 60 | HSPA5 |  |  | 373 | 0.480 | 0.2998 | Yes |
| 61 | CTSL |  |  | 376 | 0.479 | 0.3037 | Yes |
| 62 | ALDOA |  |  | 377 | 0.479 | 0.3086 | Yes |
| 63 | EIF4A2 |  |  | 380 | 0.478 | 0.3125 | Yes |
| 64 | ADAM19 |  |  | 382 | 0.477 | 0.3168 | Yes |
| 65 | COMT |  |  | 396 | 0.471 | 0.3153 | Yes |
| 66 | KITLG |  |  | 401 | 0.470 | 0.3181 | Yes |
| 67 | MAGED2 |  |  | 405 | 0.467 | 0.3214 | Yes |
| 68 | SLC3A2 |  |  | 411 | 0.466 | 0.3237 | Yes |
| 69 | GCNT2 |  |  | 413 | 0.465 | 0.3279 | Yes |
| 70 | GUCY1A2 |  |  | 435 | 0.457 | 0.3223 | Yes |
| 71 | SPRY4 |  |  | 443 | 0.453 | 0.3235 | Yes |
| 72 | DDIT3 |  |  | 447 | 0.451 | 0.3266 | Yes |
| 73 | IGF2R |  |  | 470 | 0.444 | 0.3204 | Yes |
| 74 | TCEAL8 |  |  | 471 | 0.444 | 0.3249 | Yes |
| 75 | TIPARP |  |  | 472 | 0.444 | 0.3294 | Yes |
| 76 | JUND |  |  | 478 | 0.442 | 0.3314 | Yes |
| 77 | LMO2 |  |  | 497 | 0.436 | 0.3271 | Yes |
| 78 | YBX3 |  |  | 509 | 0.431 | 0.3261 | Yes |
| 79 | LAMB2 |  |  | 524 | 0.425 | 0.3235 | Yes |
| 80 | LGALS3BP |  |  | 528 | 0.424 | 0.3264 | Yes |
| 81 | TFPI |  |  | 531 | 0.421 | 0.3297 | Yes |
| 82 | FKBP9 |  |  | 533 | 0.419 | 0.3335 | Yes |
| 83 | PARP14 |  |  | 541 | 0.417 | 0.3343 | Yes |
| 84 | GPM6B |  |  | 553 | 0.413 | 0.3331 | Yes |
| 85 | CNN3 |  |  | 576 | 0.407 | 0.3265 | Yes |
| 86 | SLC6A1 |  |  | 582 | 0.404 | 0.3281 | Yes |
| 87 | TMSB4X |  |  | 590 | 0.401 | 0.3288 | Yes |
| 88 | FOS |  |  | 592 | 0.401 | 0.3324 | Yes |
| 89 | THY1 |  |  | 593 | 0.400 | 0.3365 | Yes |
| 90 | ATP6V1G1 |  |  | 609 | 0.395 | 0.3331 | Yes |
| 91 | SLC40A1 |  |  | 612 | 0.393 | 0.3362 | Yes |
| 92 | SASH1 |  |  | 614 | 0.393 | 0.3397 | Yes |
| 93 | OXR1 |  |  | 615 | 0.393 | 0.3437 | Yes |
| 94 | IFIT2 |  |  | 624 | 0.391 | 0.3437 | Yes |
| 95 | CAV2 |  |  | 626 | 0.390 | 0.3472 | Yes |
| 96 | ARHGAP29 |  |  | 627 | 0.390 | 0.3512 | Yes |
| 97 | CHST2 |  |  | 628 | 0.390 | 0.3551 | Yes |
| 98 | NDUFB3 |  |  | 629 | 0.389 | 0.3591 | Yes |
| 99 | SNTB1 |  |  | 649 | 0.386 | 0.3537 | Yes |
| 100 | CACNB4 |  |  | 672 | 0.378 | 0.3468 | Yes |
| 101 | ADD3 |  |  | 674 | 0.378 | 0.3502 | Yes |
| 102 | BCHE |  |  | 677 | 0.376 | 0.3530 | Yes |
| 103 | EHD2 |  |  | 680 | 0.375 | 0.3558 | Yes |
| 104 | CCDC80 |  |  | 683 | 0.375 | 0.3587 | Yes |
| 105 | TNFRSF1A |  |  | 686 | 0.374 | 0.3615 | Yes |
| 106 | DNAJC3 |  |  | 689 | 0.374 | 0.3643 | Yes |
| 107 | RGS16 |  |  | 691 | 0.373 | 0.3676 | Yes |
| 108 | S100A11 |  |  | 692 | 0.373 | 0.3714 | Yes |
| 109 | SH3BGRL |  |  | 695 | 0.373 | 0.3742 | Yes |
| 110 | MEF2C |  |  | 696 | 0.372 | 0.3780 | Yes |
| 111 | CCDC102B |  |  | 702 | 0.370 | 0.3793 | Yes |
| 112 | FRMD6 |  |  | 703 | 0.370 | 0.3831 | Yes |
| 113 | TSPYL2 |  |  | 748 | 0.359 | 0.3652 | Yes |
| 114 | COQ10B |  |  | 749 | 0.359 | 0.3689 | Yes |
| 115 | AGRN |  |  | 751 | 0.359 | 0.3721 | Yes |
| 116 | COL27A1 |  |  | 760 | 0.356 | 0.3718 | Yes |
| 117 | RBMS1 |  |  | 761 | 0.355 | 0.3754 | Yes |
| 118 | MEG3 |  |  | 769 | 0.354 | 0.3756 | Yes |
| 119 | FSTL1 |  |  | 774 | 0.352 | 0.3772 | Yes |
| 120 | SLC12A2 |  |  | 782 | 0.351 | 0.3773 | Yes |
| 121 | RORA |  |  | 783 | 0.351 | 0.3809 | Yes |
| 122 | SLC38A2 |  |  | 785 | 0.350 | 0.3840 | Yes |
| 123 | TPT1 |  |  | 786 | 0.350 | 0.3875 | Yes |
| 124 | ID3 |  |  | 789 | 0.349 | 0.3901 | Yes |
| 125 | UBC |  |  | 792 | 0.348 | 0.3927 | Yes |
| 126 | SCN4B |  |  | 812 | 0.344 | 0.3869 | Yes |
| 127 | FCGRT |  |  | 814 | 0.344 | 0.3899 | Yes |
| 128 | PLEKHH2 |  |  | 822 | 0.343 | 0.3900 | Yes |
| 129 | TMEM263 |  |  | 825 | 0.342 | 0.3925 | Yes |
| 130 | MEG8 |  |  | 827 | 0.342 | 0.3955 | Yes |
| 131 | C1orf54 |  |  | 830 | 0.341 | 0.3979 | Yes |
| 132 | DAG1 |  |  | 834 | 0.340 | 0.3999 | Yes |
| 133 | ZFP36L2 |  |  | 850 | 0.336 | 0.3960 | No |
| 134 | LAPTM4A |  |  | 854 | 0.335 | 0.3980 | No |
| 135 | SEL1L |  |  | 867 | 0.332 | 0.3955 | No |
| 136 | RHOJ |  |  | 874 | 0.331 | 0.3959 | No |
| 137 | STAT3 |  |  | 880 | 0.330 | 0.3968 | No |
| 138 | CD59 |  |  | 897 | 0.326 | 0.3923 | No |
| 139 | PEAK1 |  |  | 918 | 0.323 | 0.3858 | No |
| 140 | IFI6 |  |  | 922 | 0.323 | 0.3876 | No |
| 141 | BHLHE40 |  |  | 928 | 0.321 | 0.3884 | No |
| 142 | PRKAR1A |  |  | 932 | 0.320 | 0.3902 | No |
| 143 | CD81 |  |  | 947 | 0.318 | 0.3866 | No |
| 144 | COX6A1 |  |  | 952 | 0.317 | 0.3879 | No |
| 145 | ATP6V1A |  |  | 960 | 0.316 | 0.3877 | No |
| 146 | FAT1 |  |  | 968 | 0.315 | 0.3875 | No |
| 147 | HIF1A |  |  | 972 | 0.314 | 0.3892 | No |
| 148 | EVI5 |  |  | 994 | 0.310 | 0.3821 | No |
| 149 | CD9 |  |  | 1070 | 0.297 | 0.3484 | No |
| 150 | LINGO1 |  |  | 1074 | 0.297 | 0.3499 | No |
| 151 | SPECC1 |  |  | 1089 | 0.294 | 0.3461 | No |
| 152 | RNF213 |  |  | 1091 | 0.294 | 0.3486 | No |
| 153 | LGMN |  |  | 1110 | 0.291 | 0.3427 | No |
| 154 | LAMA4 |  |  | 1114 | 0.290 | 0.3442 | No |
| 155 | LAMA2 |  |  | 1123 | 0.289 | 0.3432 | No |
| 156 | HBP1 |  |  | 1125 | 0.288 | 0.3457 | No |
| 157 | SERINC3 |  |  | 1128 | 0.288 | 0.3476 | No |
| 158 | SYPL1 |  |  | 1137 | 0.287 | 0.3466 | No |
| 159 | DMD |  |  | 1153 | 0.285 | 0.3422 | No |
| 160 | CACNB2 |  |  | 1155 | 0.285 | 0.3446 | No |
| 161 | CPM |  |  | 1160 | 0.284 | 0.3455 | No |
| 162 | CTNNB1 |  |  | 1190 | 0.280 | 0.3342 | No |
| 163 | FN1 |  |  | 1238 | 0.275 | 0.3140 | No |
| 164 | CDH11 |  |  | 1240 | 0.275 | 0.3163 | No |
| 165 | CHP1 |  |  | 1251 | 0.274 | 0.3142 | No |
| 166 | COX6C |  |  | 1252 | 0.274 | 0.3170 | No |
| 167 | NBPF14 |  |  | 1258 | 0.273 | 0.3173 | No |
| 168 | NDUFA1 |  |  | 1270 | 0.272 | 0.3147 | No |
| 169 | SBDS |  |  | 1272 | 0.272 | 0.3170 | No |
| 170 | ARHGEF7 |  |  | 1299 | 0.268 | 0.3070 | No |
| 171 | ADCY9 |  |  | 1306 | 0.268 | 0.3068 | No |
| 172 | PRRX1 |  |  | 1341 | 0.262 | 0.2928 | No |
| 173 | GAPDH |  |  | 1361 | 0.261 | 0.2862 | No |
| 174 | SLCO1C1 |  |  | 1372 | 0.260 | 0.2839 | No |
| 175 | ARHGEF12 |  |  | 1379 | 0.259 | 0.2836 | No |
| 176 | GEM |  |  | 1391 | 0.257 | 0.2809 | No |
| 177 | LBH |  |  | 1410 | 0.255 | 0.2747 | No |
| 178 | EFEMP2 |  |  | 1428 | 0.255 | 0.2689 | No |
| 179 | CALCOCO2 |  |  | 1447 | 0.253 | 0.2627 | No |
| 180 | INPP4B |  |  | 1456 | 0.251 | 0.2613 | No |
| 181 | PEX2 |  |  | 1463 | 0.251 | 0.2610 | No |
| 182 | JUNB |  |  | 1466 | 0.251 | 0.2625 | No |
| 183 | ANXA2 |  |  | 1511 | -0.263 | 0.2437 | No |
| 184 | ITGB1 |  |  | 1521 | -0.265 | 0.2420 | No |
| 185 | PSMD1 |  |  | 1532 | -0.267 | 0.2398 | No |
| 186 | PSMB6 |  |  | 1558 | -0.274 | 0.2303 | No |
| 187 | PFN1 |  |  | 1582 | -0.282 | 0.2220 | No |
| 188 | HBEGF |  |  | 1583 | -0.282 | 0.2248 | No |
| 189 | ZFP36L1 |  |  | 1590 | -0.284 | 0.2248 | No |
| 190 | APOLD1 |  |  | 1648 | -0.299 | 0.1999 | No |
| 191 | NDUFAF3 |  |  | 1656 | -0.304 | 0.1996 | No |
| 192 | KLHL23 |  |  | 1674 | -0.308 | 0.1944 | No |
| 193 | PRSS23 |  |  | 1690 | -0.316 | 0.1903 | No |
| 194 | TACC1 |  |  | 1701 | -0.319 | 0.1886 | No |
| 195 | FARP1 |  |  | 1706 | -0.321 | 0.1900 | No |
| 196 | PHLDA1 |  |  | 1718 | -0.324 | 0.1879 | No |
| 197 | GLO1 |  |  | 1755 | -0.342 | 0.1737 | No |
| 198 | LGALS1 |  |  | 1799 | -0.362 | 0.1564 | No |
| 199 | LIMS1 |  |  | 1807 | -0.365 | 0.1567 | No |
| 200 | GJC1 |  |  | 1829 | -0.379 | 0.1503 | No |
| 201 | C18orf54 |  |  | 1830 | -0.380 | 0.1541 | No |
| 202 | LDHA |  |  | 1833 | -0.380 | 0.1570 | No |
| 203 | LIMA1 |  |  | 1899 | -0.421 | 0.1295 | No |
| 204 | KDELR2 |  |  | 1925 | -0.439 | 0.1217 | No |
| 205 | CLIC1 |  |  | 1949 | -0.454 | 0.1151 | No |
| 206 | NT5DC2 |  |  | 2020 | -0.512 | 0.0861 | No |
| 207 | TPM4 |  |  | 2039 | -0.528 | 0.0826 | No |
| 208 | EPB41L2 |  |  | 2067 | -0.576 | 0.0753 | No |
| 209 | SRSF3 |  |  | 2081 | -0.606 | 0.0751 | No |
| 210 | SERPINE1 |  |  | 2161 | -0.843 | 0.0450 | No |
Table: GSEA details [plain text format]

  

Fig 2: RICHARDS\_NOWAKOWSKI\_SCIENCE\_2017\_MURAL\_UPREG: Random ES distribution      
 Gene set null distribution of ES for **RICHARDS\_NOWAKOWSKI\_SCIENCE\_2017\_MURAL\_UPREG**
